# Sample Training Based Wildfire Segmentation by 2D Histogram *θ*-Division with Minimum Error

**DOI:** 10.1155/2013/572393

**Published:** 2013-06-26

**Authors:** Jianhui Zhao, Erqian Dong, Mingui Sun, Wenyan Jia, Dengyi Zhang, Zhiyong Yuan

**Affiliations:** ^1^School of Computer, Wuhan University, Wuhan, Hubei 430072, China; ^2^Suzhou Institute of Wuhan University, Suzhou, Jiangsu 215123, China; ^3^Department of Neurosurgery, University of Pittsburgh, Pittsburgh, PA 15213, USA

## Abstract

A novel wildfire segmentation algorithm is proposed with the help of sample training based 2D histogram *θ*-division and minimum error. Based on minimum error principle and 2D color histogram, the *θ*-division methods were presented recently, but application of prior knowledge on them has not been explored. For the specific problem of wildfire segmentation, we collect sample images with manually labeled fire pixels. Then we define the probability function of error division to evaluate *θ*-division segmentations, and the optimal angle *θ* is determined by sample training. Performances in different color channels are compared, and the suitable channel is selected. To further improve the accuracy, the combination approach is presented with both *θ*-division and other segmentation methods such as GMM. Our approach is tested on real images, and the experiments prove its efficiency for wildfire segmentation.

## 1. Introduction

Image segmentation is an important research topic in the fields of image processing and computer vision, and its purpose is dividing one image into several different areas, while each region has certain characteristics and disjoints with the others. As the traditional segmentation approach, threshold value based methods [1, 2] have been widely used and studied for many years, mainly due to the fact of easy programming together with relatively low computational complexity.

The core issue of threshold methods is to construct the evaluation function to achieve the optimal segmentation threshold value. *K*-means clustering method [3] has two evaluation standards: the similarity among the elements in each subclass and the diversity among the elements in different subclasses. The main disadvantages of *K*-means method are that without prior knowledge the ideal number *K* cannot be determined before segmentation, and it is sensitive to noise and isolated targets, also sensitive to the initial clustering centers, thus is easy to obtain the local optimal solutions. Otsu method [4] is one adaptive thresholding approach. It divides the original image into two classes: object and background, according to the color histogram of image. The evaluation function is constructed based on the variance between two classes, and the best threshold value corresponds to the maximum variance. However, Otsu method ignores the spatial neighboring information of image and may fail when the size proportion of object and background is very small. Fuzzy *C*-means (FCM) [5] is also a clustering method, which promotes the target function of *C*-means with fuzzy mathematics [6]. Main advantage of FCM is using the degree of membership for classification, and thus it can conserve more information of the image and obtain better segmentation. The disadvantages of FCM are that the initial parameters are set arbitrarily, time and space complexities increase rapidly when number *C* becomes larger, and successful convergence in limited time is a problem [7]. Maximum entropy principle is used to help estimate the unknown probability distribution with limited conditions, while the main idea is to select the probability distribution with the maximum entropy value [8]. This principle is employed for image segmentation assuming that the color value of each pixel in one image is a random event, and the normalized color histogram is taken as the probability distribution of pixels [9]. The minimum error thresholding is a kind of threshold image segmentation method based on Bayesian theory [10]. It assumes that the object and background obey the mixed normal distribution, and the classification is based on the minimum error principle. It can also be explained as the minimum relative entropy between actual distribution of color histogram and assumed mixed normal distribution [11]. Through quantitative comparison [12] of the typical threshold methods, it can be found that the minimum error thresholding derives good segmentations, even for the targets with very small sizes.

**Figure 1:**
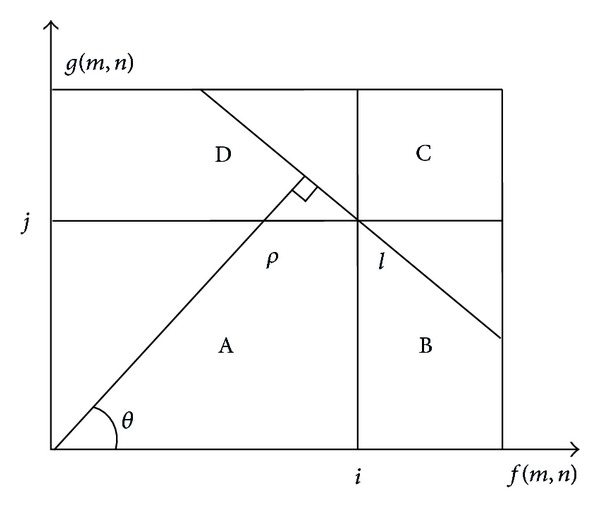
The 2D histogram with *θ*-division.

In threshold segmentation methods, color histogram is often used to describe the distribution of pixels. Since the information of spatial relationships among pixels is not considered in 1D histogram, it is very difficult to obtain satisfying segmentation results when the image has high complexity or significant noise. Therefore, 2D histogram has been employed using both color value of each pixel and its neighborhood averaged color [13–15]. As shown in Figure 1, threshold (*i*, *j*) divides the 2D histogram into 4 rectangular regions. Regions A and C represent the object and the background, respectively, while regions B and D represent edges, noise, and so on, where the probability distribution is assumed to be nearly zero. But in real images the assumption is hard to be satisfied, and thus the segmented results are less accurate. For this problem, the 2D linear division method with minimum error is introduced [16], and the line *l* perpendicular with the main diagonal line of histogram is used as threshold for segmentation. The method can divide more pixels into object or background, but it only considers the particular case; that is, angle *θ* is 45 degrees. Then a widely suitable thresholding method is proposed based on 2D histogram *θ*-division and maximum deviation criterion [17], and in this method the segmented results and running time related with different *θ* values are analyzed. More recently, new threshold method is presented based on 2D histogram *θ*-division with minimum error [18], and the influence of various *θ* values on both segmentation and computational expense is discussed with experiments.

Until now, the aforementioned threshold methods are automatic and unsupervised. Thus the segmented results cannot be affected by the prior knowledge and cannot be effectively evaluated either. Therefore, in our paper the sample training method is employed for 2D histogram *θ*-division with minimum error. Outline of our approach is to determine the suitable *θ* for specific application with sample training and to combine 2D *θ*-division with other kinds of segment techniques, for example, color ranges based methods [19, 20] such as GMM. To test our new approach, the combined algorithm is checked through segmentation of wildfire regions from some real images.

## 2. 2D Histogram and Sample Training

### 2.1. 2D Histogram *θ*-Division with Minimum Error

The 2D histogram *θ*-division with minimum error is illustrated in Figure 1, and *θ* is the angle between horizontal axis and the normal of division line *l*. When *θ* is set as 45 degrees, the weights between the pixel color and its neighboring average color are fixed at 1 : 1. When *θ* varies, the weights and the division line *l* also change, and the corresponding segmentation results are generated.

Given an image with the size of *M*∗*N*, for any pixel (*m*, *n*), its color value is *f*(*m*, *n*) and the total color level is *L*; there is 0 ≤ *f*(*m*, *n*) ≤ *L* − 1. The neighboring average color value of pixel (*m*, *n*) is

(1) $$\begin{array}{c} g\left(m,n\right)=\frac{1}{w}\sum_{(x,y)\in D}f\left(x,y\right), \end{array}$$

where *w* is the number of pixels in the neighborhood window area *D* of pixel (*m*, *n*).

Let *h*(*m*, *n*) represent the number of pixels with the color value of *f*(*m*, *n*) together with the neighboring average color value *g*(*m*, *n*), and probability of *h*(*m*, *n*) is computed as *p*(*m*, *n*) = *h*(*m*, *n*)/*M*∗*N*.

The division line *l* in Figure 1 can be expressed by

(2) $$\begin{array}{c} f\left(m,n\right)\cos\theta +g\left(m,n\right)\sin\theta =\rho , \end{array}$$

where *ρ* is the distance from the origin of coordinate to line *l*, and 0° ≤ *θ* ≤ 90°.

Based on definition of 2D color histogram, there are 0 ≤ *f*(*m*, *n*) ≤ *L* − 1, 0 ≤ *g*(*m*, *n*) ≤ *L* − 1; thus,

(3) $$\begin{array}{c} 0\leq \rho \leq \left(L-1\right)\left(\cos\theta +\sin\theta \right). \end{array}$$

To segment image, let *a* = cos⁡*θ*/(cos⁡*θ* + sin*θ*) and *T* = *ρ*/(cos⁡*θ* + sin*θ*); line *l* can be expressed with the modified formula:

(4) $$\begin{array}{c} af\left(m,n\right)+\left(1-a\right)g\left(m,n\right)=T. \end{array}$$

Therefore, variable *a* can be taken as the weighting parameter of color value *f*(*m*, *n*) and 0 ≤ *a* ≤ 1, while *T* is the corresponding segmentation threshold value. For pixel (*m*, *n*), its corresponding binarized value *b*(*m*, *n*) in the segmented image is

(5) $$\begin{array}{c} b\left(m,n\right)=\{\begin{array}{cc} 0, & af\left(m,n\right)+\left(1-a\right)g\left(m,n\right)\leq T,\\ 1, & af\left(m,n\right)+\left(1-a\right)g\left(m,n\right)>T. \end{array} \end{array}$$

Obviously, weighting variable *a* is decided by angle *θ* of division line *l*, and the segmentation result varies with different value of *a*. When *a* is more than 0.5, the color value of one pixel has heavier weight, and the boundary of segmented region is more accurate, while when *a* is less than 0.5, the neighboring average color value of pixel has heavier weight; thus segmentation's insensitivity to noise is enhanced. Therefore, segmented results can be adjusted for different applications through modifying variable *a* or angle *θ*.

The division line *l* separates one image into object area *C*_*o*_ and background area *C*_*b*_, and the corresponding probabilities are computed as *w*_*o*_(*T*) = ∑_(*i*,*j*)∈*C*_*o*__ *p*(*i*, *j*) and *w*_*b*_(*T*) = ∑_(*i*,*j*)∈*C*_*b*__ *p*(*i*, *j*).

Mean value vectors of object and background are

(6) μo(T)=[μoi(T),μoj(T)]T=[∑(i,j)∈Cop(i,j)iwo(T),∑(i,j)∈Cop(i,j)jwo(T)]T,μb(T)=[μbi(T),μbj(T)]T=[∑(i,j)∈Cbp(i,j)iwb(T),∑(i,j)∈Cbp(i,j)jwb(T)]T.

The global mean value vector is

(7) μT=[μTi,μTj]T=[∑i=0M−1∑j=0N−1p(i,j)i,∑i=0M−1∑j=0N−1p(i,j)j]T.

Variance vectors of object and background are

(8) σo2(T)=[σoi2(T),σoj2(T)]T=[∑(i,j)∈Co[i−μoi(T)]2p(i,j)wo(T)∑(i,j)∈Co[j−μoj(T)]2p(i,j)wo(T)],σb2(T)=[σbi2(T),σbj2(T)]T=[∑(i,j)∈Cb[i−μbi(T)]2p(i,j)wb(T)∑(i,j)∈Cb[j−μbj(T)]2p(i,j)wb(T)].

Based on the previous probabilities and vectors, the evaluation function of 2D histogram thresholding with minimum error is defined as

(9) J(T)=1−wo(T)ln⁡wo(T)−wb(T)ln⁡wb(T)+wo(T)ln⁡[σoi(T)σoj(T)]+wb(T)ln⁡[σbi(T)σbj(T)].

When *J*(*T*) obtains its minimum value, there is the following corresponding optimal threshold value T* [16–18]:

(10) $$\begin{array}{c} T^{*}=\underset{0\leq T\leq L-1}{\arg\;\min}J\left(T\right). \end{array}$$

**Figure 2:**
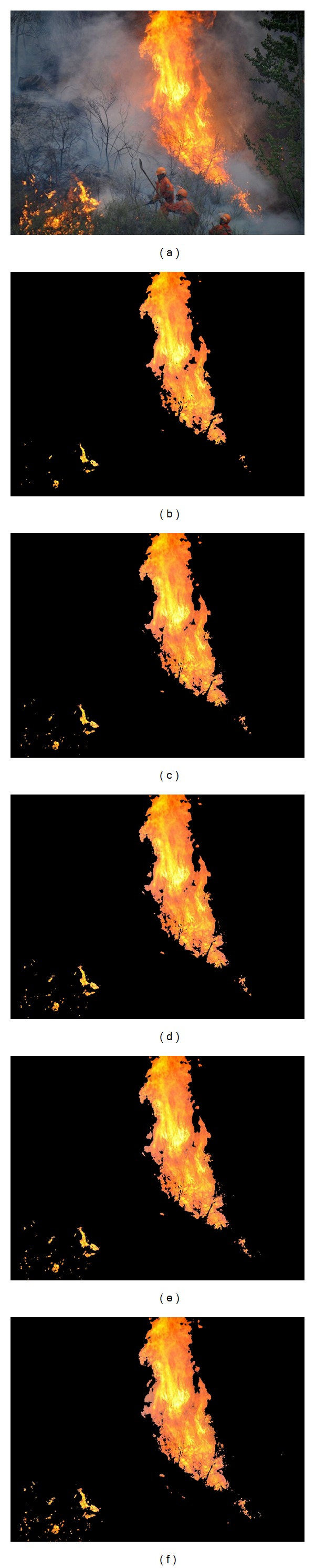
Fire segmentation with different angles *θ*: (a) input image; (b–f) results with *θ* = 0°, 30°, 45°, 60°, 90°.

To illustrate the effects of angle *θ* on the segmented results, one image with wildfire is used. Taking the V channel of HSV color space as input for 2D histogram *θ*-division with minimum error, the segmentations with different angle *θ* are shown in Figure 2.

From the results of Figure 2, effects of angle *θ* on the segmentation can be found. When *θ* is small, most noises are deleted but some fire pixels are also discarded. When *θ* is large, most fire pixels are detected while some noises are falsely taken as flame too. Therefore, determining the optimal value of angle *θ* is very important.

### 2.2. Sample Training Based *θ* Determination

**Figure 3:**
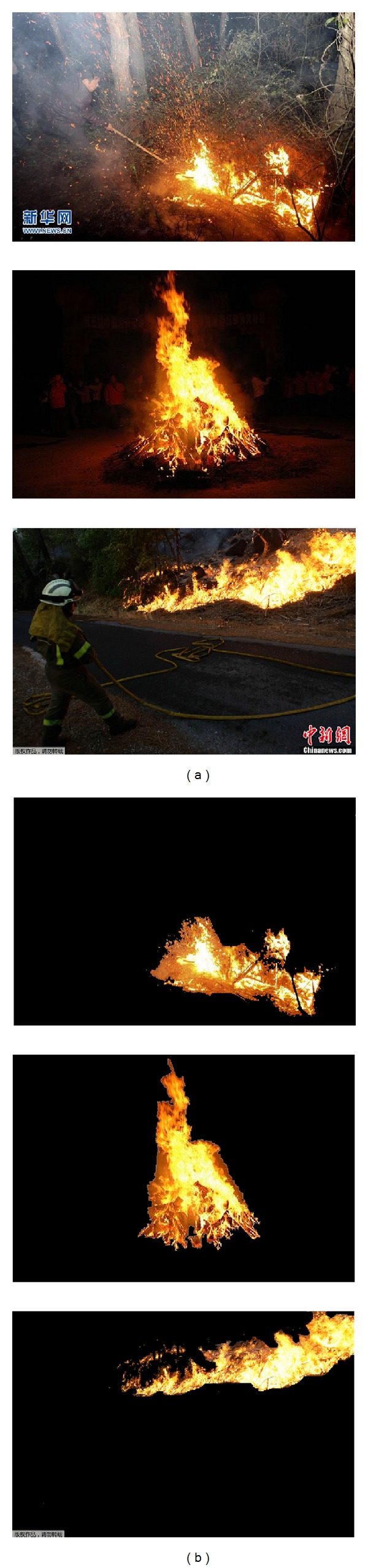
Sample images and manually marked fires.

The 2D histogram *θ*-division with minimum error is an unsupervised threshold method, thus the application of prior knowledge helps to obtain better results. For the specific problem of fire segmentation, the optimal angle *θ* can be determined through sample learning. As shown in Figure 3, typical images with wildfire are collected, and the fire regions are marked manually, which are used to evaluate and train the segmentation method.

For any sample image (Figure 3(a)), we mark the background area with pure black color (value 0) and leave the object area unchanged, and the manual template image *r* is thus generated (Figure 3(b)). For the same sample image, 2D histogram division method with angle *θ* and minimum error is used for segmentation and the segmented image *s* is produced. For one pixel with coordinates (*x*, *y*), there are 4 possible cases as follows:

1. if *r*(*x*, *y*) = *s*_*θ*_(*x*, *y*) > 0, the pixel has been correctly identified as the object point;
2. if *r*(*x*, *y*) = *s*_*θ*_(*x*, *y*) = 0, the pixel has been correctly identified as the background point;
3. if *r*(*x*, *y*) = 0 and *s*_*θ*_(*x*, *y*) > 0, the pixel has been wrongly identified as the object point;
4. if *r*(*x*, *y*) > 0 and *s*_*θ*_(*x*, *y*) = 0, the pixel has been wrongly identified as the background point.

Based on comparison between image *r* and image *s*, we can obtain the total number of pixels, the number of object pixels, the number of background pixels, the number of pixels that are wrongly identified as object, and the number of pixels that are wrongly identified as background. Then the segmented result can be evaluated by the probability of error division as follows:

(11) $$\begin{array}{c} P_{\mathrm{err}}\left(\theta \right)=P\left(O\right)\cdot P\left(B\;|\;O\right)+P\left(B\right)\cdot P\left(O\;|\;B\right), \end{array}$$

where *P*(*O*) and *P*(*B*) are probabilities of object and background, respectively, *P*(*O* | *B*) is probability that background is wrongly identified as object, *P*(*B* | *O*) is probability that object is falsely identified as background, and the probabilities can be computed by aforementioned numbers of different kinds of pixels.

Obviously the probability of error division *P*_*err*⁡_(*θ*) is a function of angle *θ*, and the minimum function value is related with the optimal angle *θ*.

**Figure 4:**
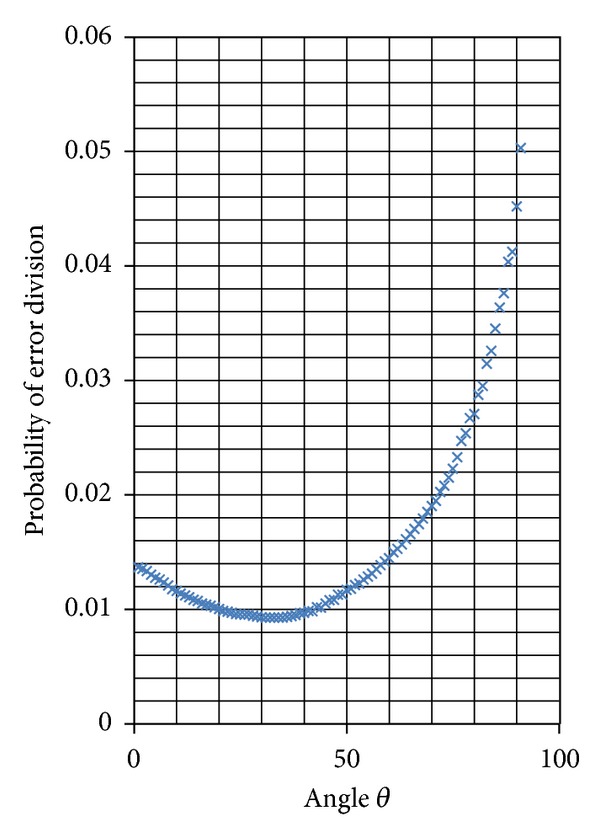
Relationship between *P*_*err*⁡_(*θ*) and angle *θ*.

With a group of wildfire images having total 1767705 pixels and manually marked fire regions having 114320 pixels, relationship between *P*_*err*⁡_(*θ*) value and angle *θ*, varying in the range of [0°, 90°] with the changing step of 1°, is shown in Figure 4.

The optimal angle *θ* corresponds with the minimum value of *P*_*err*⁡_(*θ*). Since distribution of discrete sampled points in Figure 4 approximates the shape of one unimodal function, the method of bisection search is used to find the minimum value of *P*_*err*⁡_(*θ*). For the example shown in Figure 4, the optimal angle *θ* is found as *θ* = 32.9375° and the related minimum error probability of evaluation function *P*_*err*⁡_(*θ*) is 0.0093.

## 3. Application on Wildfire Segmentation

### 3.1. Selection of Color Channel

The 2D histogram *θ*-division with minimum error can deal with gray image or any channel of color image. To analyze its performance on different color channels, both RGB and HSV color models are used for testing, and all color channels together with gray images of three color fire images are processed.

**Figure 5:**
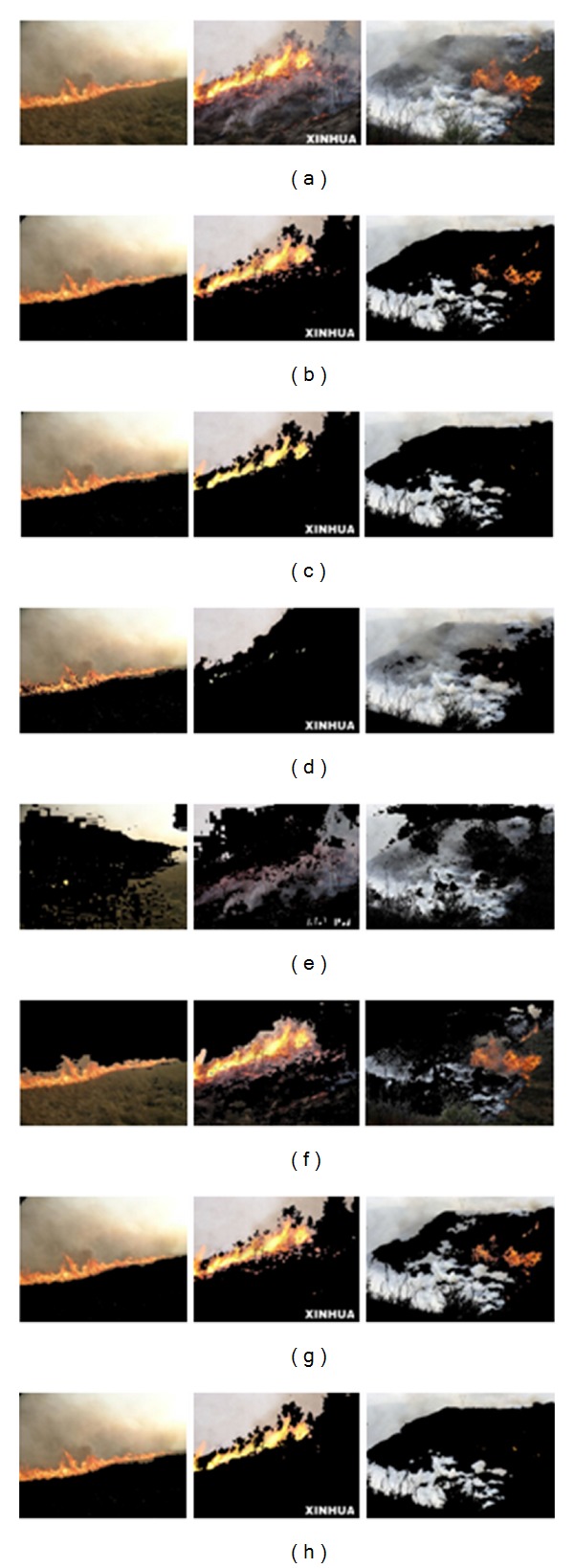
Results on color channels and gray images—the 1st row: input images; the 2nd row to 8th row: segmentations of R, G, B, H, S, V channels and gray images.

The segmented results are shown in Figure 5; (a) shows input images, (b) shows results of R channel, (c) shows results of G channel, (d) shows results of B channel, (e) shows results of H channel, (f) shows results of S channel, (g) shows results of V channel, and (h) shows results of gray images which are converted from the input color images.

For wildfire images, the regions of interest are fire and smoke; thus the evaluation of color channels is based on whether there are successful segmentations of the regions from images. For G channel or B channel, the fire or the smoke regions cannot be identified correctly. R channel can obtain fire regions more accurately, but there are still some fire pixels wrongly classified as nonfire objects, while the segmented smoke regions are less accurate. H channel has worse results, since hue differences among real fire, smoke and background are not very obvious; for example, hue of dark fire and smoke is similar with background. S channel derives most fire regions but can also take some saturated pixels of background as fire. V channel has the ability to identify smoke and fire areas as high luminance objects at the same time. Comparatively, segmentations from gray images lose some fire regions.

It can be found that no channel successfully segments fire or smoke region separately, but V channel has the best performance to obtain both fire and smoke areas simultaneously. Therefore, V channel is selected for 2D histogram *θ*-division with minimum error. Of course, to obtain the individual objects of fire or smoke, segmented results need further process, for example, the popularly employed color range based segmentation methods.

### 3.2. Combination with Other Segmentation Methods

**Figure 6:**
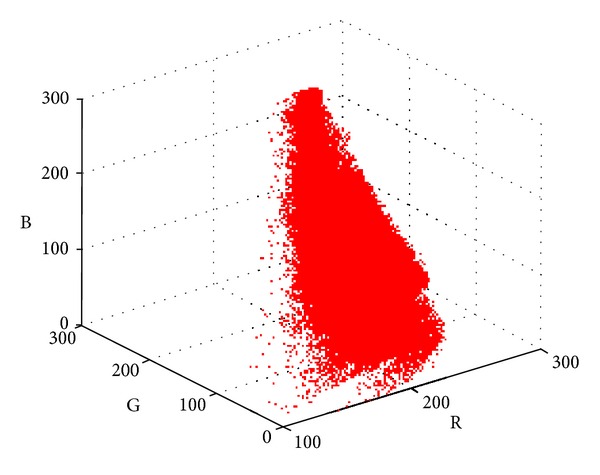
Sample pixels used for GMM training.

There are other kinds of methods for fire segmentation from images; among them the Gaussian mixture model (GMM) for sample distribution is an efficient one. In our previous work [20], 530,000 pixels manually segmented from the fire regions of 23 sample images are collected in RGB color space (shown in Figure 6), and then the 3D shape of fire sample pixels is represented by GMM. Parameters of GMM are trained with expectation maximization, and then the fire probability distribution in 3D color space is computed. Based on the calculated fire probability of one pixel under processing, we can decide whether it belongs to flame area or not.

GMM parameters and models are represented as

(12) g(x,μi,Σi)  =1(2π)d|Σi|exp⁡⁡(−12(x−μi)TΣi−1(x−μi)),p(x)=Σiαi∗g(x,μi,Σi),

where the weighting value, kernel center, and covariance matrix of one single Gaussian model are *α*_*i*_, *μ*_*i*_, and Σ_*i*_, respectively, while *p*(*x*) illustrates how close of point *x* to the Gaussian mixture model. The suitable number of Gaussian models in GMM can be manually assigned or computed automatically.

**Figure 7:**
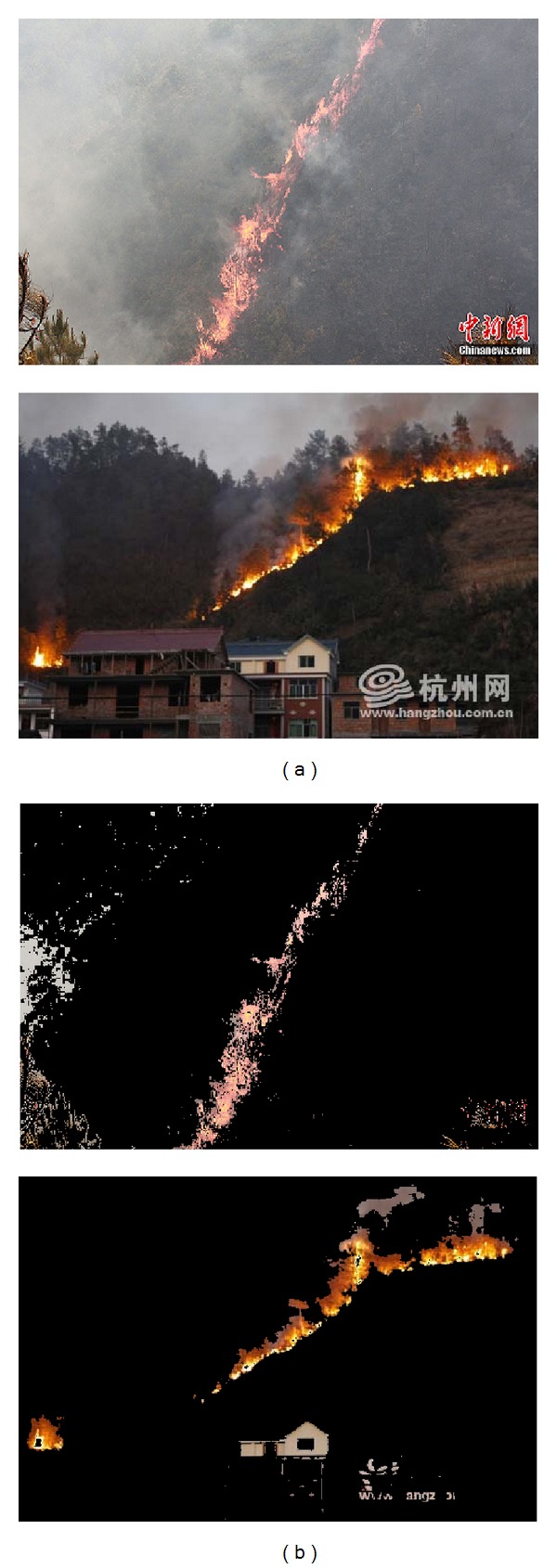
Segmented results from GMM with errors.

However, performance of GMM is affected by the amount of sample pixels and the number of Gaussian models. If the Gaussian models or the sample pixels are not enough, GMM cannot precisely describe the object range in color space. In this case, application of GMM on the input image easily brings errors, for example, wrongly taking some nonfire objects as fires. As shown in Figure 7, figures of the 1st column are input images, while figures of the 2nd column are the segmented results. Having the similar color as fire, some nonfire pixels, such as smoke, wall, or words, are mistakenly detected as flame.

Therefore, combination of the 2D histogram *θ*-division with minimum error and other division methods such as GMM can further improve the segmentation. There are 2 possible ways for combination: (1) 2D *θ*-division first and then GMM segmentation; (2) GMM segmentation first and then 2D *θ*-division.

For (1), GMM deals with the already segmented fire and smoke areas from 2D *θ*-division; thus the pixels of other objects have no chance to be mistakenly chosen as flame. For (2), 2D *θ*-division method further divides the segmented result from GMM, which makes it possible to differentiate fire pixels and nonfire similar pixels again in images of the 2nd column of Figure 7.

Taking (1) combination as example, the basic steps of our algorithm using both 2D histogram *θ*-division with minimum error and GMM are the following.

**Step 1**

Determine the optimal angle *θ* based on sample training for wildfire detection.

**Step 2**

Segment the input image using 2D histogram *θ*-division with minimum error.

**Step 3**

Obtain wildfire regions with GMM processing from the segmented results of Step 2.

## 4. Results and Discussion

**Figure 8:**
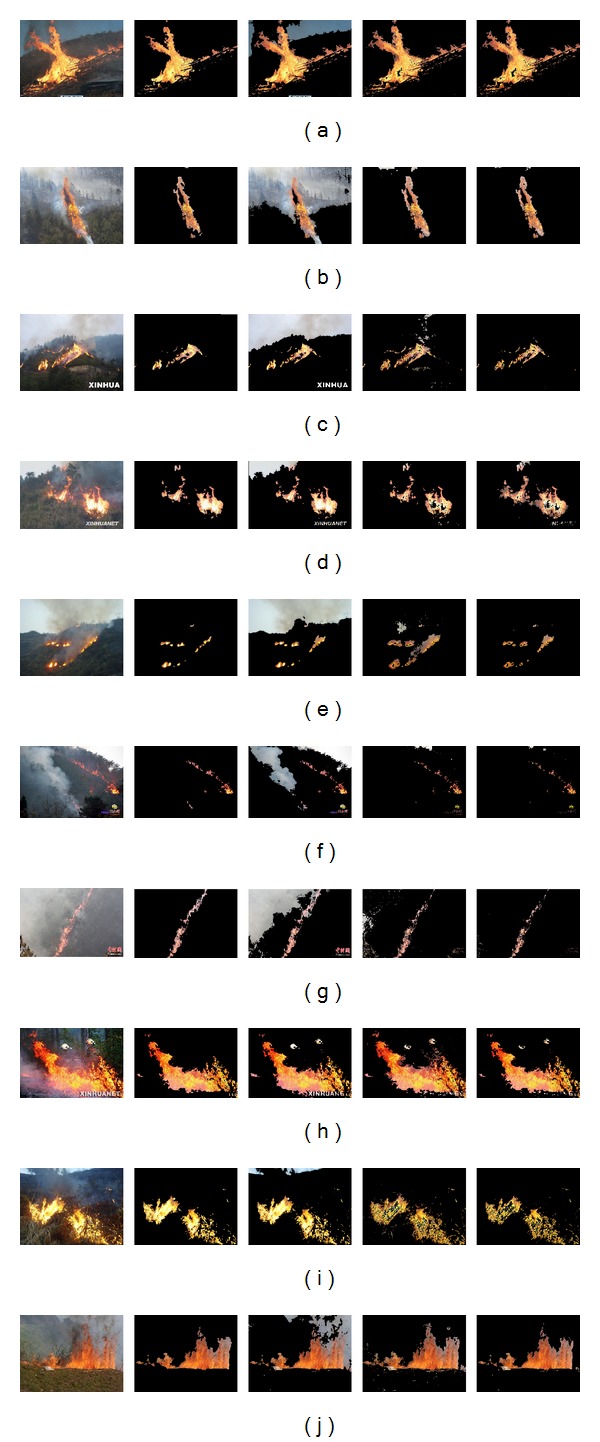
Experimental results of wildfire segmentation from images.

More real pictures with various wildfires are used for experiments, and the results are displayed in Figure 8. The 1st column shows input images; the 2nd column shows manually marked fire pixels used for comparison; the 3rd column shows segmented results from the 2D histogram *θ*-division (*θ* = 45°, untrained angle) with minimum error; the 4th column shows segmented results using only GMM; the 5th column shows final results of the combined algorithm, that is, first 2D histogram *θ*-division (*θ* = 32.9375°, the trained optimal angle) with minimum error and then GMM.

From the experimental results, it can be found that the segmentation using only GMM still has the problem of wrongly taking non-fire pixels as fires. With the help of 2D histogram *θ*-division with minimum error and the sample trained optimal angle *θ*, most of the non-fire pixels are filtered; that is, our combined algorithm finally obtains better results of wildfire regions.

The computational expense for segmentation is also tested with a computer of Intel Core2 Duo CPU 2.93 GHz, 2 GB RAM. For the combined algorithm running on 10 pictures of Figure 8 with already trained angle *θ* and GMM parameters, their computational costs are listed in Table 1, where the unit of time is the second.

It can be found that the computational expense of our combined algorithm is not high, making it possible to be used in real applications with fire recognition. Having the ability of processing more than 1 frame per second, the method can work for video based approaches.

## 5. Conclusion

Based on 2D color histogram and the minimum error principle, *θ*-division methods have been studied in recent years. The segmented results are affected by angle *θ*, but its relationship with prior knowledge of certain problems still needs further study. Taking fire as a typical example, the sample training based 2D histogram *θ*-division with minimum error is implemented, and its application for segmentation is explored and analyzed.

Segmentation from angle *θ* is evaluated by the defined probability function of error division. Through collected samples and training, the optimal angle *θ* is found. Color channels are compared and the suitable one is chosen.

Then a new combined approach with 2D *θ*-division and GMM method is proposed to derive more accurate segmenting results. Compared with using only GMM, the combined method helps filter more errors. Our method has low computing cost and can be used for video based approaches with the help of temporal information.

The proposed techniques are verified with some real images, and the computing expenses are also tested. The combined algorithm obtains satisfying results, which has proved the ability of sample training base 2D histogram *θ*-division with minimum error. In the future, the presented methods will be further verified with the other kinds of segmentation techniques and will be tried for the other kinds of applications besides fire detection.

## Figures and Tables

**Table 1:** Computing cost of the combined algorithm.

Image no.	Resolution	Time
Figure 8(a)	410 × 308	0.578
Figure 8(b)	519 × 389	0.400
Figure 8(c)	350 × 263	0.475
Figure 8(d)	480 × 318	0.438
Figure 8(e)	410 × 308	0.245
Figure 8(f)	410 × 285	0.487
Figure 8(g)	460 × 307	0.427
Figure 8(h)	450 × 305	0.839
Figure 8(i)	492 × 369	0.605
Figure 8(j)	300 × 225	0.269
